# Green Synthesis of Chromium Oxide Nanoparticles for Antibacterial, Antioxidant Anticancer, and Biocompatibility Activities

**DOI:** 10.3390/ijms22020502

**Published:** 2021-01-06

**Authors:** Shakeel Ahmad Khan, Sammia Shahid, Sadaf Hanif, Hesham S. Almoallim, Sulaiman Ali Alharbi, Hanen Sellami

**Affiliations:** 1 Center of Super-Diamond and Advanced Films (COSDAF), Department of Chemistry, City University of Hong Kong, 83 Tat Chee Avenue, 999077 Kowloon, Hong Kong; 2 Department of Chemistry, School of Science, University of Management and Technology, Lahore 54770, Pakistan; Sadafhanif59@gmail.com; 3 Department of Oral and Maxillofacial Surgery, College of Dentistry, King Saud University, P.O. Box-60169, Riyadh 11545, Saudi Arabia; hkhalil@ksu.edu.sa; 4 Department of Botany and Microbiology, College of Science, King Saud University, PO Box-2455, Riyadh 11545, Saudi Arabia; sharbi@ksu.edu.sa; 5 Laboratory of Treatment and Valorization of Water Rejects, Water Research and Technologies Center (CERTE), Borj-Cedria Technopark, University of Carthage, Soliman 8020, Tunisia; sellami_hanen@yahoo.fr

**Keywords:** green synthesis, Cr_2_O_3_, *Abutilon indicum* (L.) Sweet, antibacterial, anticancer, antioxidant, biocompatibility

## Abstract

This study deals with the green synthesis of chromium oxide (Cr_2_O_3_) nanoparticles using a leaf extract of *Abutilon indicum* (L.) Sweet as a reducing and capping agent. Different characterization techniques were used to characterize the synthesized nanoparticles such as X-ray diffraction (XRD), Scanning electron microscope (SEM), Transmission electron microscope (TEM), Energy-dispersive X-ray (EDX), Fourier transform infrared (FTIR), X-ray photoelectron spectroscopy (XPS), and ultraviolet-visible (UV-VIS) spectroscopy. The X-ray diffraction technique confirmed the purity and crystallinity of the Cr_2_O_3_ nanoparticles. The average size of the nanoparticles ranged from 17 to 42 nm. The antibacterial activity of the green synthesized nanoparticles was evaluated against four different bacterial strains, *E. coli*, *S. aureus*, *B. bronchiseptica*, and *B. subtilis* using agar well diffusion and a live/dead staining assay. The anticancer activities were determined against Michigan Cancer Foundation-7 (MCF-7) cancer cells using MTT and a live/dead staining assay. Antioxidant activity was investigated in the linoleic acid system. Moreover, the cytobiocompatibility was analyzed against the Vero cell lines using MTT and a live/dead staining assay. The results demonstrated that the green synthesized Cr_2_O_3_ nanoparticles exhibited superior antibacterial activity in terms of zones of inhibition (ZOIs) against Gram-positive and Gram-negative bacteria compared to plant extracts and chemically synthesized Cr_2_O_3_ nanoparticles (commercial), but comparable to the standard drug (Leflox). The green synthesized Cr_2_O_3_ nanoparticles exhibited significant anticancer and antioxidant activities against MCF-7 cancerous cells and the linoleic acid system, respectively, compared to chemically synthesized Cr_2_O_3_ nanoparticles. Moreover, cytobiocompatibility analysis displayed that they presented excellent biocompatibility with Vero cell lines than that of chemically synthesized Cr_2_O_3_ nanoparticles. These results suggest that the green synthesized Cr_2_O_3_ nanoparticles’ enhanced biological activities might be attributed to a synergetic effect. Hence, green synthesized Cr_2_O_3_ nanoparticles could prove to be promising candidates for future biomedical applications.

## 1. Introduction

Nanobiotechnology is the intersection of biology and nanotechnology that deals with nanotechnology’s application in different biological systems. Nanobiotechnology further deals with the fabrication of biocompatible, ecofriendly, and biogenic nanomaterials and nanoparticles [1]. The nanoparticle Cr_2_O_3_, is of high significance and interest among various metal oxides-based nanoparticles because of its unique physicochemical properties such as a wide bandgap (~3.4 eV), high melting temperature, and increased stability [2]. The Cr_2_O_3_ nanoparticles have been widely utilized in different applications, including catalysis, photonics, coating materials, advanced colorants, etc. [3,4,5,6]. The trivalent Cr_2_O_3_ nanoparticles are considered the most stable compared to other chromium oxides [7]. Despite being a promising material, few studies have evaluated Cr_2_O_3_ nanoparticles for different biological applications because of their potential toxic effects that have been reported in many studies [8]. The biocompatibility of Cr_2_O_3_ nanoparticles is an essential parameter for their utilization in different biological systems. The poisonous effects of Cr_2_O_3_ nanoparticles can be reduced by coating or functionalization their surfaces with biogenic materials. One of the most promising ways of achieving this, is surface coating Cr_2_O_3_ with plants’ biogenic phytomolecules [9].

The synthesis of nanoparticles using plants as a precursor has attracted much attention recently. As an alternative to conventional chemical and physical methods, the green synthesis of nanoparticles using biological sources (plants) is an economical, robust, ecofriendly, and easily scalable technique [10]. Most importantly, nanoparticles synthesized using plants appear to be more biocompatible than those prepared with chemical and physical methods. This is because of the fact that toxic chemicals are used in traditional chemical and physical techniques for synthesizing nanoparticles. After several rounds of washing, these toxic chemicals cannot easily be removed from the nanoparticle’s surface. Therefore, poisonous chemicals present on the nanoparticle’s surface making them less biocompatible and limiting their biological applications. Instead, plant based green synthesis of nanoparticles uses phytomolecules as the reducing and capping agents, and no additional chemicals are required. Moreover, plant biogenic phytomolecules have molecular functionalities that are biologically active and have antibacterial, antioxidant, anticancer, etc. properties. So, green synthesis using plants enhances the nanoparticle’s biocompatibility and is responsible for the synergetic effect [9,10].

In this work, we synthesized Cr_2_O_3_ nanoparticles using leaf extracts of a medicinal plant (*Abutilon indicum* (L.) Sweet) for the first time, as per the author’s best knowledge. *Abutilon indicum* (L.) Sweet has been widely employed for treating different kinds of diseases in Tamils, Siddha, Chinese, and traditional Ayurvedic medicine [10,11,12]. *Abutilon indicum* (L.) Sweet is a rich source of different biogenic phytomolecules such as terpenoids, alkaloids, saponins, polyphenols, tannins, etc., with various biological applications [13]. Many useful and biologically active compounds have been isolated from leaf extracts of *Abutilon indicum* (L.) Sweet [14]. Many reports are available that highlight the biological importance of this plant [15]. Till now, many plants have been utilized for the synthesis of nanoparticles. Among the plants used, some are either not biologically active or they are biologically active but have toxic effects. Therefore, nanoparticles for biological applications need to be synthesized with such plants that are biologically active with no toxic effects. In this regard, *Abutilon indicum* (L.) Sweet appeared as a more prominent plant that has both of these properties compared to other plants [10]. Many nanomaterials such as nanoparticles (gold, silver, ZnO, etc.) and nanorods (1D-MoO_3_, etc.) have also been synthesized using leaf extracts of *Abutilon indicum* (L.) Sweet [11,12,16]. We have previously reported the green synthesis of MnO and CuO using leaf extracts of *Abutilon indicum* (L.) Sweet [10,17]. In this study, we have further utilized this plant for the green synthesis of Cr_2_O_3_ nanoparticles. The synthesized Cr_2_O_3_ nanoparticles using leaf extracts of *Abutilon indicum* (L.) Sweet were further evaluated for antibacterial, anticancer, biocompatibility, and antioxidant activities. They have presented excellent antioxidant and anticancer activities. The synthesized nanoparticles exhibited outstanding antibacterial activity by inhibiting the growth of both Gram-positive and Gram-negative bacterial strains. Moreover, the green synthesized Cr_2_O_3_ nanoparticles demonstrated excellent biocompatibility compared to chemically synthesized and already reported Cr_2_O_3_ nanoparticles.

## 2. Results and Discussion

### 2.1. Characterization

**Figure 1:**
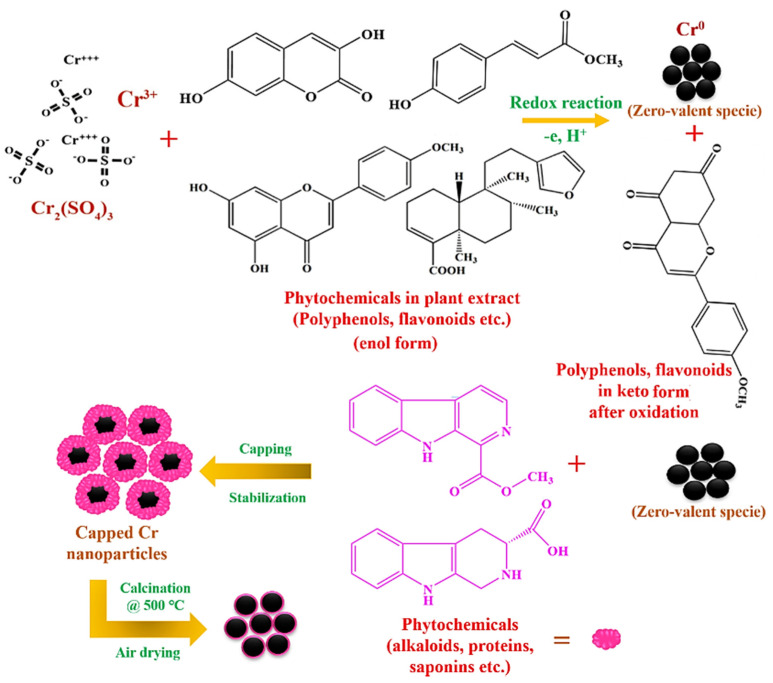
The schematic presentation for the green synthesis of Cr_2_O_3_ nanoparticles using *Abutilon indicum* (L.) Sweet leaf extract.

*Abutilon indicum* (L.) Sweet leaf extract was used as a reducing and capping agent for the synthesis of Cr_2_O_3_ nanoparticles. The Cr_2_O_3_ nanoparticles synthesis was monitored visually by detecting color change upon the addition of metal salt precursor in leaf extract. The color change of the reaction mixture from red to black indicated the formation of desired nanoparticles. This color transition occurred due to the surface plasmon resonance (SPR) phenomenon on the nanoparticle’s surface [2,18]. *Abutilon indicum* (L.) Sweet leaf extract contains a rich source of biologically active phytomolecules (polyphenols, flavonoids, terpenoids, alkaloids, tannins, saponins, proteins, etc.) [10,13,19]. These phytomolecules can act as ligands and chelate with different metal ions to reduce and stabilize their ions to nano form [20,21]. The chromium sulfate salt (Cr_2_(SO_4_)_3_), upon dissolution in water, becomes a freely moving ion. The freely moving Cr^3+^ ions due to electron-deficiency are attracted towards the plant’s phytomolecules (polyphenols, etc.). As a result of this, chelate complex formation occurs between metal ions and the plant’s phytomolecules upon transferring electrons (donor–acceptor mechanism) from oxygen to Cr^3+^ (Figure 1) [20,21]. This leads to the oxidation of polyphenols, flavonoids, etc., and converts them into keto form (Figure 1). On the other hand, Cr^3+^ is reduced to zero-valent specie Cr^0^ and simultaneously stabilized by the other plant’s phytomolecules (alkaloids, flavonoids, tannins, etc.) present in their vicinity. During air-drying and calcination, they are readily oxidized and converted into Cr_2_O_3_ nanoparticles capped with phytomolecules of *Abutilon indicum* (L.) Sweet leaf extract [20,21,22]. A similar green synthesis mechanism was also reported to synthesize ZnO, zinc oxide–silver, Fe_3_O_4_, and magnetite (Fe_3_O_4_) using different plants by Khalafi et al., Gurgur et al., López et al., and Yew et al., respectively [20,21,22,23].

**Figure 2:**
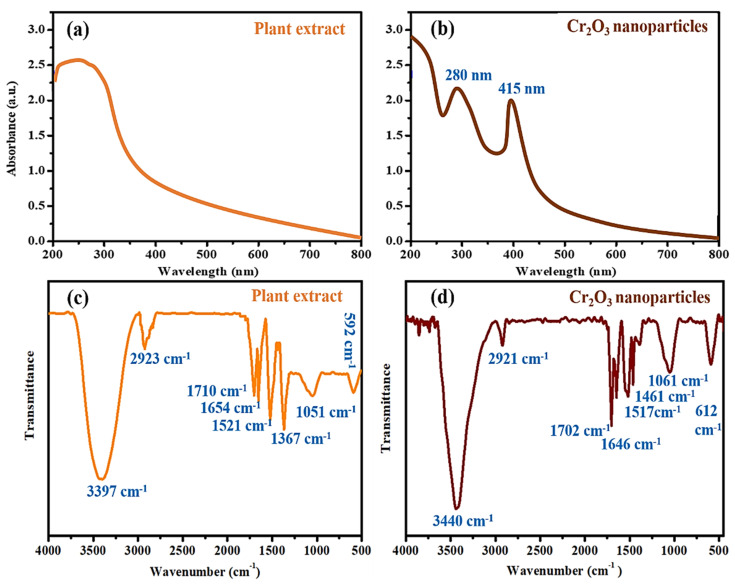
UV–Visible spectra of (**a**) plant leaf extract, (**b**) Cr_2_O_3_ nanoparticles. FTIR of (**c**) plant extract and (**d**) Cr_2_O_3_ nanoparticles.

Further, the green synthesized Cr_2_O_3_ nanoparticles were analyzed using a UV–Visible spectrophotometer, and the results are presented in Figure 2a,b. UV–Visible spectrum results indicated the presence of two absorption peaks at 280 nm and 415 nm. The absorption band in the UV region is attributed to phytomolecules such as polyphenols and flavonoids, and these molecules absorb UV light because of the OH moieties [13,24,25]. The absorption band in the visible region corresponds to Cr_2_O_3_ [26,27,28,29]. Moreover, the plant leaf extracts presented the UV region’s absorption band (200–390 nm) [10]. The FTIR analysis was further performed to determine the phytomolecules involved in synthesizing Cr_2_O_3_ nanoparticles as reducing and capping agents. Figure 2b,c presented the FTIR spectrum of plant leaf extract and nanoparticles. The results demonstrated that the synthesized nanoparticles displayed different FTIR peaks corresponding to O-H (3430 cm^−1^), C-H (2921 cm^−1^), C=O (1702 cm^−1^), N-H (1646 cm^−1^), C=C (1517 cm^−1^), and C-O-C (1061 cm^−1^). These peaks are matched with the FTIR signals of the leaf extracts with slight shifting. These results suggest that many biologically active phytomolecules are left adsorbed on the surface of the Cr_2_O_3_ nanoparticle [9,10]. Moreover, the FTIR signal at 612 cm^−1^, corresponding to Cr-O, further validated the metal-oxygen bond formation [2,7,18].

**Figure 3:**
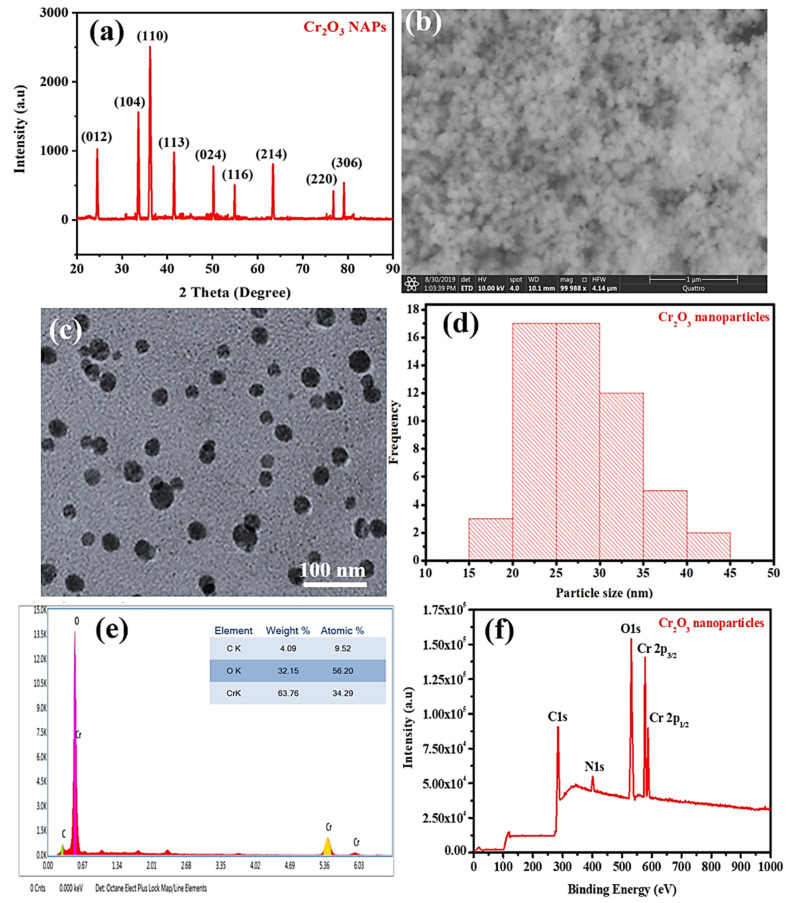
(**a**) XRD, (**b**) SEM, scale bar = 1 µm, (**c**) TEM, (**d**) DLS particle size distribution, (**e**) EDX and (**f**) XPS analysis of green synthesized Cr_2_O_3_ nanoparticles.

The crystallinity of the green synthesized Cr_2_O_3_ nanoparticles was determined by XRD analysis, and the results are presented in Figure 3a. The XRD spectrum of synthesized nanoparticles revealed nine different Bragg’s diffraction peaks, indexing to crystal planes of (012), (104), (110), (113), (024), (116), (214), (220), and (306) at 2ϴ = 24.5°, 33.6°, 36.2°, 41.5°, 50.2°, 54.9°, 63.4°, 76.8°, and 79.1°, respectively. The diffraction peaks of Cr_2_O_3_ nanoparticles are well-matched with Joint Committee on Powder Diffraction Standards (JCPDS) 38–1479 [30,31]. The peaks associated with impurities were not observed, indicating the purity of the nanoparticles. The peak’s intensity further displayed the high crystalline nature of the nanoparticles. Figure 3b,c shows the SEM and TEM images of the synthesized Cr_2_O_3_ nanoparticles. SEM and TEM images displayed that the synthesized nanoparticles have spherical morphology. The Cr_2_O_3_ nanoparticles size determined by TEM ranged from 35–60 nm. The average nanoparticle size determined using DLS was 27.76 nm and ranged from 17–42 nm (Figure 3d). TEM and DLS particle size analysis results are consistent with each other.

The compositional analysis of the synthesized nanoparticles was performed using Energy-dispersive X-ray spectroscopy. EDX spectra results showed that the nanoparticles were mainly composed of chromium (63.76%) and oxygen (32.15%), as shown in Figure 3e [2]. One extra peak associated with carbon (4.09%) is also evident in the EDX spectrum. The carbon peak could be attributed to the presence of phytomolecules (polyphenols, alkaloids, flavonoids, etc.) of leaf extract of *A. indicum* (L.) Sweet adsorbed on Cr_2_O_3_ nanoparticle’s surface [9,10].

Further, the elemental analysis was also carried out using X-ray photoelectron spectroscopy (XPS), and the results are presented are shown in Figure 3f. XPS spectrum results indicated the presence of five peaks at binding energies of 284.5, 400.9, 530.9, 576.9, and 586.8 eV. These XPS peaks correspond to C1s, N1s, O1s, Cr2p_3/2_, and Cr2p_1/2_, respectively [32,33]. The carbon and nitrogen XPS peaks, other than oxygen and chromium, might be attributed to the adsorption of phytomolecules of *Abutilon indicum* (L.) Sweet leaf extract on the surface of nanoparticles. The phytomolecules of *Abutilon indicum* (L.) Sweet leaf extract has different molecular functionalities such as -OH, -NH_2_, -CHO, -CHO_2_, etc., in their molecules [13]. Both EDX and XPS analysis results are found to be consistent with each other. All these characterization results corroborated that the Cr_2_O_3_ nanoparticles of interest have been successfully green synthesized.

### 2.2. Antibacterial Propensity

**Figure 4:**
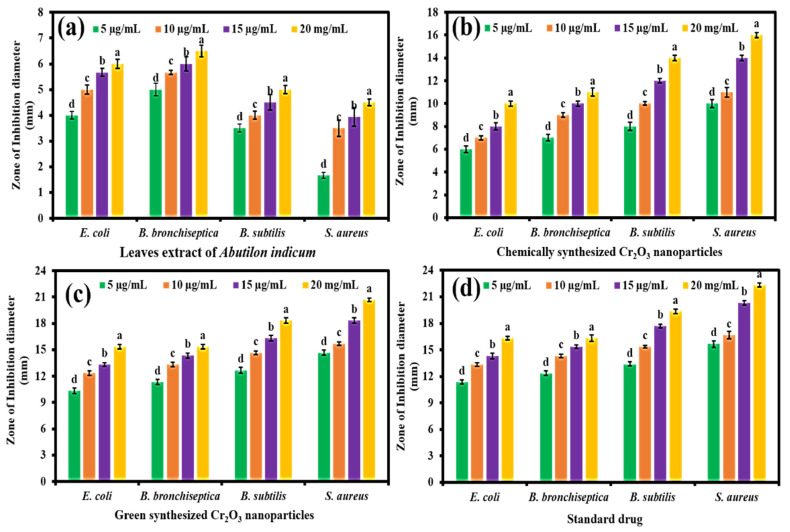
The antibacterial activity of (**a**) *Abutilon indicum* (L.) Sweet leaf extract, (**b**) chemically synthesized Cr_2_O_3_ nanoparticles, (**c**) green synthesized Cr_2_O_3_ nanoparticles in terms of zones of inhibition (ZOIs) at different concentration levels against different bacteria compared to standard drug (**d**). (Note: Tukey based heterogeneous lower-case letters represent significant pairs).

The green synthesized Cr_2_O_3_ nanoparticles were evaluated for their antibacterial potential compared to the plant extract, chemically synthesized Cr_2_O_3_ nanoparticles, and the standard drug against four different pathogenic bacteria, including two Gram-positive and two Gram-negative. The results showed that all the samples presented concentration-dependent antibacterial activity, and the maximum inhibition in the bacteria’s growth was observed with a 20 µg/mL concentration (Figure 4a–d). Moreover, the green synthesized Cr_2_O_3_ nanoparticles exhibited superior antibacterial activity in terms of ZOIs against Gram-positive and Gram-negative bacteria compared to the plant extract and chemically synthesized Cr_2_O_3_ nanoparticles. At all the concentration levels, they presented comparable inhibitory efficacy compared to the standard drug. The results further demonstrated that Gram-positive bacteria were found to be more susceptible than Gram-negative bacteria towards the green synthesized Cr_2_O_3_ nanoparticles. This might be due to the variances in the chemical structure and composition of both the bacteria’s cell wall, and further, their different level of susceptibility towards metal oxide nanoparticles. The cell wall of Gram-negative bacteria is composed of lipopolysaccharides, lipoproteins, and phospholipids. In contrast, Gram-positive bacteria’s cell walls include a thin layer of peptidoglycan and teichoic acid and large pores. Moreover, compared with Gram-negative bacteria, Gram-positive bacteria have a high negative charge on the cell wall surface, attracting nanoparticles more efficiently. Hence, the small size of nanoparticles at low temperatures can penetrate, spread, and damage the bacterial cell wall, which leads to bacteria demise [34].

**Figure 5:**
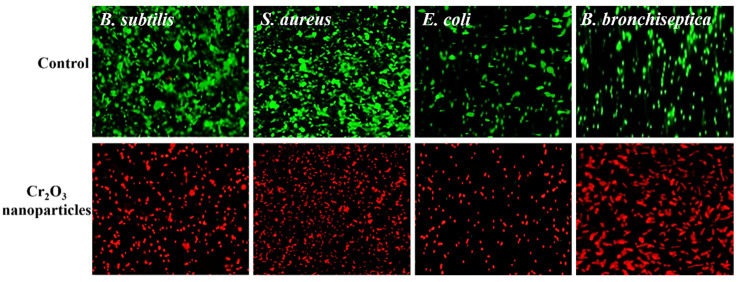
Live/dead bacterial cell images—live cells stained with green (SYTO-9) while dead cells stained with red (PI) (scale bar = 50 µm).

The antibacterial activity of the green synthesized nanoparticles was further confirmed by CLSM, and the results are presented in Figure 5. SYTO-9 is a membrane-permeant dye which stains live/dead cells. In comparison, PI is an impermeant dye and can only stain dead cells upon its penetration. The PI penetrates the cells only via the dead cells’ burst membrane and subsequently binds to the DNA, emitting a strong red fluorescence [35]. The results demonstrate that untreated bacterial cells (control) exhibited an intense green color, indicating that all the cells were alive and intact. On the other hand, bacterial cells treated with green synthesized Cr_2_O_3_ nanoparticles appeared red, which showed that the nanoparticles destroyed the bacterial cell’s membrane’s permeability and integrity, leading to cell demise.

### 2.3. Anticancer Activity

**Figure 6:**
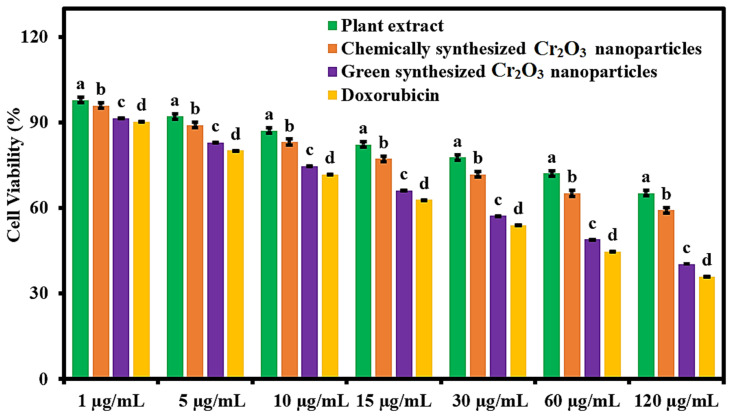
Anticancer activity of green synthesized Cr_2_O_3_ nanoparticles in terms of cell viability percentage against MCF-7 cancer cells compared to *Abutilon indicum* (L.) Sweet leaf extract, chemically synthesized Cr_2_O_3_ nanoparticles, and standard drug. (Note: Tukey based heterogeneous lower-case letters represent significant pairs).

The green synthesized Cr_2_O_3_ nanoparticles were evaluated for their anticancer potential in terms of cell viability percentage against MCF-7 cancerous cells compared to *Abutilon indicum* (L.) Sweet leaf extract, chemically synthesized Cr_2_O_3_ nanoparticles, and the standard drug. The results were shown that all the samples presented concentration-dependent anticancer activity. The maximum cytotoxic effect on MCF-7 cancer cells was observed with 120 µg/mL concentration of all the samples (Figure 6). The superior anticancer activity was demonstrated by the green synthesized Cr_2_O_3_ nanoparticles compared to *Abutilon indicum* (L.) Sweet leaf extract and chemically synthesized Cr_2_O_3_ nanoparticles at all tested concentrations. Moreover, the green synthesized Cr_2_O_3_ nanoparticles displayed slightly less anticancer activity against MCF-7 carcinoma cells than the standard drug. However, this difference was not sufficient, so we can suggest that they presented comparable levels of anticancer activity to the standard drug at all the tested concentrations. Our green synthesized Cr_2_O_3_ nanoparticles displayed better anticancer activity against human breast cancer cells at the 120 µg/mL concentration compared to (500 µg/mL) single-phase Cr_2_O_3_ nanoparticles synthesized using *Nephelium lappaceum* L. [36].

**Figure 7:**
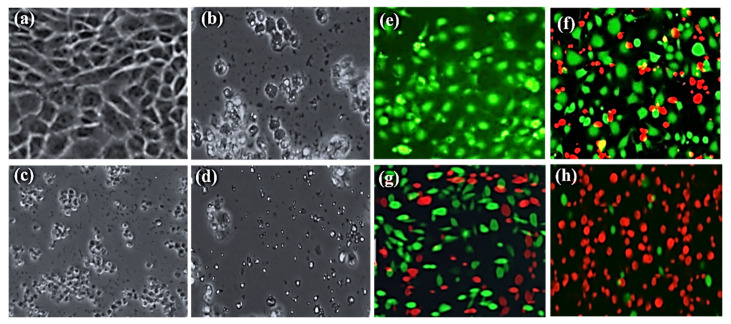
The morphological alterations in MCF-7 cancer cells after treatment with (**b**) plant extract, (**c**) chemically synthesized Cr_2_O_3_ nanoparticles, and (**d**) green synthesized Cr_2_O_3_ nanoparticles. The live/dead MCF-7 cancer cells stained with green and red fluorescent dye respectively after treatment with (**f**) plant extract, (**g**) chemically synthesized Cr_2_O_3_ nanoparticles, and (**h**) green synthesized Cr_2_O_3_ nanoparticles. (**a**) and (**e**) controls. (Scale bar = 100 µm).

Using an inverted microscope (Nikon Eclipse TE200), we further observed the morphological changes in MCF-7 carcinoma cells after their treatment with green synthesized Cr_2_O_3_ nanoparticles, plant extract, and chemically synthesized Cr_2_O_3_ nanoparticles at the concentration of 120 µg/mL. Figure 7a–d shows the inverted micrograph of MCF-7 cancerous cells. The images show that after treatment, drastic changes occurred in the morphology of MCF-7 cancer cells. The MCF-cells’ volume and cytoplasm have been decreased, and the shape of the cells changed to round. All the samples induced toxicity, but green synthesized Cr_2_O_3_ nanoparticles were appeared to pose a significant and severe cytotoxic effect on MCF-7 cancer cells.

To further affirm the anticancer activity against MCF-7 cancer cells, the live and dead fluorescence staining assay was employed using CLSM. Figure 7e–h shows the live/dead MCF-7 cancer cells stained with green and red dye, respectively. The results demonstrated that green synthesized Cr_2_O_3_ nanoparticles exhibited a maximum cytotoxic effect on MCF-7 carcinoma cells, and they had killed almost 90% cancerous cells (Figure 7h). On the other hand, chemically synthesized Cr_2_O_3_ nanoparticles induced a mild toxic effect on MCF-7 cancer cells and destroyed almost 50% of cancer cells. It is interesting to note that leaf extract also presented toxicity on MCF-7 cancer cells indicating that *Abutilon indicum* (L.) Sweet has biologically active phytomolecules. Henceforth, these results are consistent with the results of MTT and inverted microscopic analysis.

### 2.4. Antioxidant Activity

**Figure 8:**
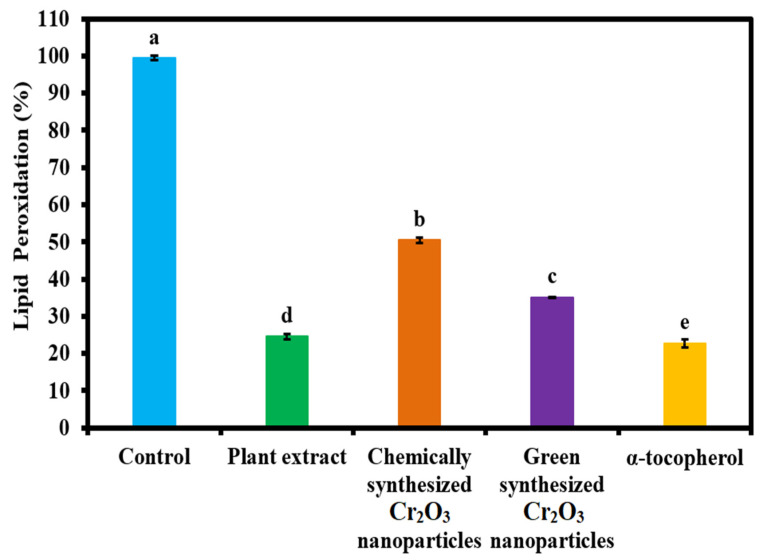
The antioxidant activity of green synthesized Cr_2_O_3_ nanoparticles in terms of linoleic acid peroxidation percentage compared to plant extract, chemically synthesized Cr_2_O_3_ nanoparticles, and standard (α-tocopherol). (Note: Tukey based heterogeneous lower-case letters represent significant pairs).

The antioxidant activity of green synthesized Cr_2_O_3_ nanoparticles was determined in the linoleic acid system and compared to plant leaf extract, chemically synthesized Cr_2_O_3_ nanoparticles, and standard (α-tocopherol). The results in the form of lipid peroxidation percentage are presented in Figure 8. The results demonstrate that maximum lipid peroxidation inhibition was observed with the standard, followed by plant extract and green synthesized Cr_2_O_3_ nanoparticles. Chemically synthesized Cr_2_O_3_ nanoparticles displayed the lowest level of antioxidant activity in terms of lipid peroxidation inhibition. It is interesting to note that the plant extract presented a comparable antioxidant activity compared to the standard. The enhanced antioxidant activity of green synthesized Cr_2_O_3_ nanoparticles might be attributed to the presence of phytomolecules of the plant leaf extract on the nanoparticle’s surface, as evident from the FTIR, EDX, and XPS results. Hence, these results suggest that green synthesized Cr_2_O_3_ nanoparticles and plant extract can be used as powerful antioxidant agents in different applications. Moreover, our green synthesized Cr_2_O_3_ nanoparticles appeared to be more active in terms of antioxidant activity than previously reported for Cr_2_O_3_ nanoparticles synthesized using leaf extract of *Rhamnus virgate* [2].

### 2.5. Cytobiocompatibility Analysis

**Figure 9:**
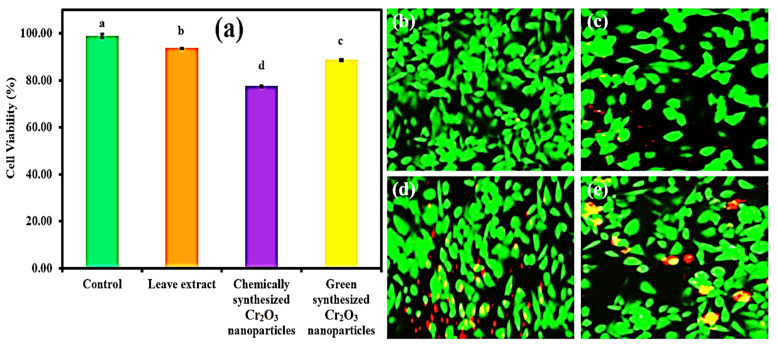
(**a**) The cytobiocompatibility analysis of green synthesized Cr_2_O_3_ nanoparticles against Vero cell lines compared to plant extract and chemically synthesized Cr_2_O_3_ nanoparticles. (Note: Tukey based heterogeneous lower-case letters represent significant pairs). CLSM images of (**b**) untreated Vero cell lines (control), and treated with (**c**) plant extract, (**d**) chemically synthesized Cr_2_O_3_, and (**e**) green synthesized Cr_2_O_3_ nanoparticles (Live cells with green and dead cells with red) (Scale bar = 50 µm).

The green synthesized Cr_2_O_3_ nanoparticles were further evaluated for their cytobiocompatibility analysis against the Vero cell lines (Kidney epithelial cells) in comparison to plant leaf extract and chemically synthesized Cr_2_O_3_ nanoparticles. As per International Organization for Standardization (ISO) 10993-5, a material could be considered toxic, moderately toxic, weak toxic, and cytobiocompatible if the cell viability (%) is less than 40%, 40 to 60%, 60 to 80%, and greater than 80% respectively. The results are presented in Figure 9a. The results of cell viability (%) demonstrated that the chemically synthesized Cr_2_O_3_ nanoparticles exhibited the least cytobiocompatibility (77.46 ± 0.31%). On the other hand, plant extract and green synthesized Cr_2_O_3_ nanoparticles presented excellent cytobiocompatibility (93.63 ± 0.24% and 88.50 ± 0.85%) with the Vero cell lines, respectively. Our green synthesized Cr_2_O_3_ nanoparticles exhibited good cytobiocompatibility with the normal cells compared to previous reports [2].

We further analyzed the cytobiocompatibility of the green synthesized Cr_2_O_3_ nanoparticles with Vero cell lines compared to the plant extract and chemically synthesized Cr_2_O_3_ nanoparticles using the live/dead staining technique. The results are displayed in Figure 9b–e. The results demonstrated that the plant leaf extract and green synthesized Cr_2_O_3_ nanoparticles exerted the lowest levels of cytotoxic effects on Vero cells as fewer cells died (Figure 9c,e). In contrast, chemically synthesized Cr_2_O_3_ nanoparticles exerted more cytotoxicity, and many cells appeared dead (Red) (Figure 9d). The good cytobiocompatibility of the green synthesized Cr_2_O_3_ nanoparticles with the Vero cell lines might be attributed to the presence of phytomolecules of plant leaf extract.

## 3. Materials and Methods

The present research work was performed in the chemistry laboratory, Department of Chemistry, University of Management and Technology, Lahore. All the chemicals used were of analytical grade and available commercially. The chemicals used in the research work were purchased from Merck (Darmstadt, Germany) and Sigma Chemicals Co. (St. Louis, MS, USA). The commercially available Oleic acid-coated Cr_2_O_3_ nanoparticles were purchased with a 18 nm size for comparative biological analysis—these nanoparticles are named as chemically synthesized nanoparticles.

### 3.1. Collection of the Plant Material

*Abutilon indicum* (L.) Sweet plant was collected from the wild area native to tropical and subtropical regions. Its identification was made by Dr. Zaheer (Department of Botany, Punjab University, Lahore, Pakistan).

### 3.2. Preparation of Plant Extract

**Figure 10:**
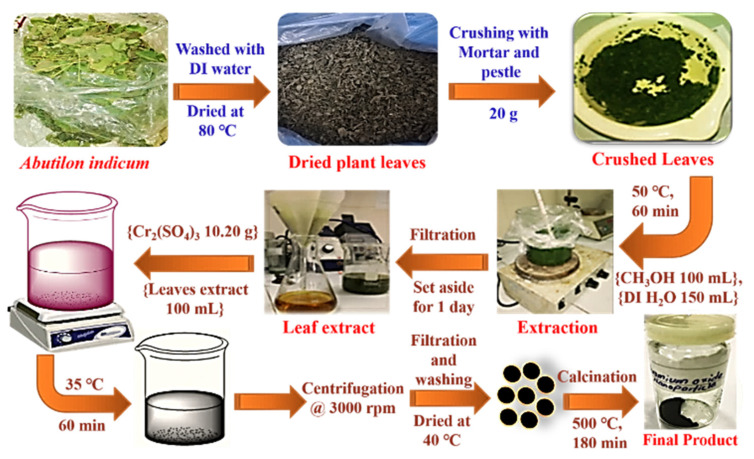
The leaf extraction preparation and green synthesis of Cr_2_O_3_ nanoparticles using *Abutilon indicum* (L.) Sweet leaf extracts.

A total of 20 g of the plant’s fresh leaves were taken. The leaves were washed with deionized (DI) water and dried in an oven at 80 °C. The dried leaves were crushed with a mortar and pestle. After fine crushing, the crushed leaves were mixed in 150 mL DI water and 100 mL methanol and heated at 50 °C for 1 h. After heating, the sample was kept for one day and then filtered, and the subsequent filtrate (plant extract) was stored in an air-tight bottle for further use (Figure 10).

### 3.3. Green Synthesis of Chromium Oxide (Cr_2_O_3_) Nanoparticles

For the green synthesis of Cr_2_O_3_ nanoparticles, 10.20 g of Cr_2_(SO_4_)_3_ was added to 100 mL of plant extract and stirred the resultant mixture for 60 minutes at 35 °C. After, a change in the color of the resulting mixture solution from red to black was observed, due to surface plasmon resonance indicating the formation of the required Cr_2_O_3_ nanoparticles. The nanoparticles were then centrifuged at 3000 rpm twice and then filtered and washed with deionized water/ethanol three times. Then, the Cr_2_O_3_ nanoparticles were dried in an oven at 40 °C and further calcinated at 500 °C in a muffle furnace for 3 hours (Figure 10). Finally, the obtained nanoparticles were stored in an air-tight container for characterization and biological applications.

### 3.4. Characterization

#### 3.4.1. X-ray Diffraction

The crystallinity and purity of the green synthesized Cr_2_O_3_ nanoparticles in powder form were determined using the powder X-ray diffraction (XRD) (Bruker D2 PHASER with LYNXEYE XE-T detector, Haidian, Beijing, China) with a wavelength (λ) of 0.154 nm over the 2θ range 4–90°.

#### 3.4.2. Scanning Electron Microscope (SEM) and Energy-Dispersive X-ray (EDX) Spectroscopy 

The synthesized nanoparticles’ morphology was characterized using an SEM (Quattro S) by placing the dried powder sample on the carbon tape. The compositional analysis was carried out with an energy-dispersive X-ray (EDX) spectroscopy using Thermo Fisher Scientific Ultradry (Madison, WI, USA) attached with SEM.

#### 3.4.3. Transmission Electron Microscope (TEM)

The green synthesized Cr_2_O_3_ nanoparticles were dissolved in methanol, and sonication was performed at 25–30 °C and then they were transferred to a copper grid. The copper grid was set aside for drying for 5–10 min, then TEM (FEI/Philips Tecnai 12 BioTWIN, Baltimore, MD, USA) was used to acquire TEM images with an acceleration voltage of 200 kV [37].

#### 3.4.4. Zetasizer Dynamic Light Scattering (DLS)

The green synthesized Cr_2_O_3_ nanoparticles were dissolved in DI water and sonicated for 5 min at 25–30 °C. About 10 mm sample solution was taken out and placed on glass cuvette. After that, the cuvette was placed in the cell holder, and scanning was performed using a dynamic light scattering particle size analyzer (Malvern Zetasizer Nano ZS, Worcestershire, WR14 1XZ, UK) from 1 to 100 nm at 25–30 °C [37].

#### 3.4.5. X-ray Photoelectron Spectroscopy (XPS)

The XPS of green synthesized Cr_2_O_3_ nanoparticles was performed using ULVAC-PHI Quantera II (Ulvac-PHI Inc., Chigasaki. Kanagawa, Japan) with the following conditions monochromatic AlKα (hυ = 1486.6 eV) at 25.6 W with a beam diameter of 100 µm. Additionally, pass energy of 280 eV with 1 eV per step was used to perform a wide scan analysis.

#### 3.4.6. UV-Visible Spectrophotometric Analysis

The green synthesized Cr_2_O_3_ nanoparticles were dissolved in DI water and sonicated for 5 min at 25–30 °C. The nanoparticles solution and plant extract were then transferred in a quartz cuvette, and after being placed in the cell, the absorption maxima were determined from 200 to 800 nm using a UV–Visible spectrophotometer (Shimadzu 1700, Columbia, Maryland, U.S.A.) at 25–30 °C [37].

#### 3.4.7. Fourier Transform Infrared (FTIR)

The dried powder of green synthesized Cr_2_O_3_ and plant extract was placed on the quartz slide, and then the FTIR spectrum was measured from 450 to 4000 using Perkin Elmer Spectrum 100 spectrophotometer (Bridgeport Avenue Shelton, CT 06484-4794, USA) at 25–30 °C.

### 3.5. Antibacterial Propensity

The antibacterial propensity of the green synthesized Cr_2_O_3_ nanoparticles was determined using agar well diffusion assay against four different bacterial strains (*Staphylococcus aureus* ATCC^®^ 23235™, *Bacillus subtilis* ATCC^®^ 6051™, *Escherichia coli* ATCC^®^ 25922™, and *Bordetella bronchiseptica* ATCC^®^ 4617™), following the protocol previously reported by [9]. The antibiotic drug (Leflox) and dimethyl sulfoxide (DMSO) were used as a positive and negative control, respectively. Four concentrations (5, 10, 15, and 20 µg/mL) of each sample were prepared in DMSO. For antibacterial activity, washed petri-dishes and freshly synthesized media (nutrient agar) were sterilized by an autoclave for 15 min at 121 °C. The sterilized molten nutrient agar (30 mL) was poured into petri-dishes as a basal layer and set aside for a moment to form a solid gel, and subsequently, 3.5 mL of inoculum of each bacterium inoculated. The inoculum of each bacteria strain was prepared at 1 × 10^8^ CFU/mL. The holes were then bored at four peripheral positions using a sterilized hollow iron rod. The holes were then filled with 20 µL of sample, positive and negative control dilutions. The petri-dishes were incubated for 24 h at 37 °C in an incubator. After 24 h, the clear zones of inhibition (ZOIs) were observed around the holes. The diameter of the ZOIs was recorded using a ruler in millimeters. The experiment was repeated three times.

#### Live/dead Bacteria Staining Assay

To further confirm the antibacterial activity of green synthesized Cr_2_O_3_ nanoparticles, a live and staining assay was performed using a confocal laser scanning microscope (CLSM, FV-1200, Olympus, Tokyo, Japan) following the protocol reported by [38]. The two fluorescent dyes SYTO-9 and propidium iodide (PI) were used for staining the live (green) and dead (red) bacteria, respectively. Each bacterium was cultured in a nutrient broth and incubated at 37 °C for 24 h to obtain the confluence of 10^5^–10^6^ colony forming units (CFU) per mL. After, bacteria were inoculated onto pasteurized cover glass coated with poly-L-lysine in 24-microtitre well plate and placed in an incubator for incubation for 1 hour to allow bacterial cells to attach to the cover glass. The suspended bacterial cells were then removed, and the cover glass was gently rinsed three times using a saline solution. Each bacterium on the cover glass was treated with 20 µg/mL concentration of green synthesized Cr_2_O_3_ nanoparticles and incubated again at 37 °C for 24 h. The bacteria on cover glass were then stained with a live/dead bacterial viability kit, as per the manufacturer’s instructions. The bacterial cells were analyzed with CLSM at 485 nm excitation wavelength for SYTO-9 and PI and 530 nm emission wavelength for SYTO-9 while 630 nm for PI. We only considered green synthesized Cr_2_O_3_ nanoparticles for live/dead staining assay as they presented excellent antibacterial properties in terms of ZOIs.

### 3.6. Anticancer Activity

MTT (3-(4,5-dimethylthiazol-2-yl)-2,5-diphenyltetrazolium bromide) colorimetric assay was used for determining the anticancer activity of chromium oxide nanoparticles against MCF-7 (breast cancer cells) [10]. The MCF-7 carcinomatous cells were placed in Dulbecco’s Modified Eagle’s Medium (DMEM) in a humidified atmosphere consisting of 5% CO_2_ and 95% air at 37 °C. To obtain the 5 × 10^8^ cells/well, the MCF-7 cells were cultured in 96-well plates containing the 100 µL of DMEM for 24 h at 37 °C. The 50 µL of each sample (green synthesized Cr_2_O_3_ nanoparticles, plant extract, and pristine) at concentrations of 1, 5, 10, 15, 30, 60, and 120 µg/mL was added separately in each well, and the plate was then incubated for 24 h at 37 °C. After the plate was centrifuged to remove the supernatant and then washed with phosphate buffer saline. The 15 microliters of MTT reagent (0.5 mg/mL) was added to each well. The plate was placed in an incubator for incubation for 4 hours at 37 °C. To dissolve the crystals of formazan, a reduced product of MTT, 150 µL of DMSO were added in each well and stirred on a shaker for 10 min. The optical density (OD) of formazans products was measured at 570 nm using a spectrophotometer. The cell viability percentage was calculated using the following formula; with the help of the following equation;

% Cell viability = OD_sample_/OD_control_ ×100 (1)

#### Live/Dead Cells Staining Assay

We further investigated the MCF-7 carcinomatous cell viability with the fluorescent staining technique to affirm the cytotoxicity using the live/dead double staining kit (viable cells stain with green and dead cells with red). The same experiment was repeated as described above till cancer cells treated with different samples (10 µL of 120 μg/mL) and subsequent incubation. After, the staining solution (4 μg/mL) was added to each well at 37 °C and incubated for 20 min. Photographs were taken with a fluorescence microscope (excitation wavelength 488/545 nm for viable/dead cells).

### 3.7. Antioxidant Activity in Terms of Linoleic Acid (%) Inhibition

The antioxidant activity of the green synthesized Cr_2_O_3_ nanoparticles in terms of linoleic acid (%) inhibition was determined compared to plant extracts and chemically synthesized Cr_2_O_3_ nanoparticles, following the protocol reported by [39]. In detail, 100 µg/mL concentration of each sample was added to the solution mixture of 0.2 M sodium phosphate buffer (pH 7.0, 10 mL), 99.99% ethanol (10 mL), and linoleic acid (0.13 mL). The resulting solution’s total volume was made up to 25 mL with DI water and subsequently incubated for 360 hours at 40 °C. The extent of oxidation was measured using the thiocyanate method. Accordingly, 0.2 mL of the sample solution was taken and then added to 10 mL of ethanol (75%). Subsequently, 0.2 mL of aqueous ammonium thiocyanate solution (30%) and 0.2 mL FeCl_2_ (20 mM in 3.5% HCl) was added. The reaction mixture was stirred for 3 min, and the absorption maxima were then measured at 500 nm wavelength. The percentage inhibition of linoleic acid was calculated using the following formula:

% Inhibition = [100 − (Absorbance of sample)/(absorbance of control)] × 100(2)

The alpha-tocopherol was used as an external standard, and the control only consisted of linoleic acid without any treatment.

### 3.8. Cytobiocompatibility Analysis

The green synthesized Cr_2_O_3_ nanoparticles were further evaluated for their cytobiocompatibility analysis against the Vero cell line (Kidney epithelial cells) in comparison to chemically synthesized Cr_2_O_3_ nanoparticles. The MTT protocol was followed for determining the cytobiocompatibility analysis reported by [40].

### 3.9. Statistical Analysis

All the experiments (antibacterial, anticancer, antioxidant, and biocompatibility) were conducted three times, and the results are presented as mean ± standard deviation. One-way ANOVA at a fixed significance level (0.05) and the Tukey test were also applied to the results to determine the significance.

## 4. Conclusions

In this work, Cr_2_O_3_ nanoparticles have been successfully green synthesized using the leaf extract of *Abutilon indicum* (L.) Sweet as a reducing and capping agent. The green synthesized Cr_2_O_3_ nanoparticles were successfully characterized using XRD, SEM, TEM, EDX, FTIR, XPS, and UV-VIS spectroscopy. The green synthesized Cr_2_O_3_ nanoparticles displayed excellent antibacterial performance against all tested bacterial strains (*E. coli, S. aureus, B. bronchiseptica*, and *B. subtilis*) and were comparable to the standard available drugs. However, they showed better bacterial inhibition than plant leaf extract and chemically synthesized Cr_2_O_3_ nanoparticles. The green synthesized Cr_2_O_3_ nanoparticles also demonstrated significant anticancer and antioxidant activities against MCF-7 cancer cells and the linoleic acid system, respectively, comparable to the employed standard drug and external standard antioxidant, respectively. Moreover, green synthesized Cr_2_O_3_ nanoparticles presented excellent biocompatibility with Vero cell lines compared to chemically synthesized Cr_2_O_3_ nanoparticles. It is interesting to note that *Abutilon indicum* (L.) Sweet leaf extract was also found to be active towards antibacterial, antioxidant, and anticancer activities. These results suggest that the green synthesized Cr_2_O_3_ nanoparticles’ enhanced biological activities might be attributed to the synergetic effect (physical properties and adsorbed phytomolecules on their surface). Thus, the antioxidant, antibacterial, biocompatibility, and anticancer activities results displayed the potential of green synthesized Cr_2_O_3_ nanoparticles for different future biomedical applications (antifungal, antilarvicidal, etc.). Hence, nanoparticles synthesis using leaf extracts of *Abutilon indicum* (L.) Sweet is an efficient, robust, economical, and green method that produces biocompatible and biological active nanoparticles. The use of leaf extracts of *Abutilon indicum* (L.) Sweet can be further extended for synthesizing various other biocompatible nanomaterials for biological applications.

## Data Availability

The data are not publicly available.
